# Assessment of the GOES-16 Clear Sky Mask Product over the Contiguous USA Using CALIPSO Retrievals

**DOI:** 10.3390/rs12101630

**Published:** 2020-05-20

**Authors:** Pedro A. Jiménez

**Affiliations:** National Center for Atmospheric Research, Boulder, CO 80301, USA

**Keywords:** GOES-16, CALIPSO, ACM, NWP, cloud initialization, MAD-WRF model

## Abstract

Cloud initialization is a challenge in numerical weather prediction. Probably the most relevant observations for this task come from geostationary satellites. These satellites provide the cloud mask with high spatio-temporal resolution and low latencies. The low latency is an attractive option for nowcasting systems such as the solar irradiance nowcasting model MAD-WRF. In this study we examine the potential of using the cloud mask from the GOES-16 satellite over the contiguous U.S. for this particular application. With this aim, the GOES-16 cloud mask product is compared against CALIPSO retrievals during a two year period. Both the GOES-16 data and the CALIPSO retrievals are interpolated to a grid that covers the contiguous U.S. at 9 km of horizontal grid spacing that is being used in MAD-WRF nowcasts. Results indicate a probability of detection, or accuracy, of all sky conditions of 86.0%. However, the accuracy is higher for cloud detections, 90.9% than for clear sky detections 74.8%. The lower performance of clear sky retrievals is a result of missdetections during daytime. This is especially clear for summer, and for regions to the north of parallel 36 during winter. However, regions to the south of parallel 36 show acceptable performance during both daytime and nighttime. It is over these regions wherein the cloud mask product should show its largest potential to enhance the cloud initialization in the MAD-WRF model.

## 1. Introduction

The first step in numerical weather prediction (NWP) is to have an accurate estimation of the atmospheric state to initialize the NWP model. Probably the most challenging aspect in this step is to estimate the characteristics of the cloud field: location of the clouds and their vertical extent, as well as the type/content of the hydrometeors. The challenge arises from either inaccuracies in the observations or inadequate spatio-temporal sampling for NWP applications. The problem is aggravated for nowcasting applications wherein the latency of the data could become an issue. A clear example is nowcasting surface irradiance for solar energy applications e.g., [1,2] wherein misplacement of clouds during the initialization can introduce large errors even in the very short term predictions.

An attractive option to initialize the cloud field in nowcasting applications is to use retrievals from geostationary satellites. The retrievals from these satellites have low latencies (minutes) and adequate spatio-temporal samplings for NWP applications. For instance, the Advanced Baseline Imager ABI, [3] on board of the GOES-R satellites provides full disk scans every 10 minutes at a resolution ranging from 0.5 to 2 km depending on the spectral band. In addition, the spectral resolution is now comparable with the one from instruments on board of circumpolar satellites such as the Moderate Resolution Imaging Spectroradiometer (MODIS) or the Visible Infrared Imaging Radiometer Suite (VIIRS) which should translate into more accurate retrievals of the cloud variables than the previous GOES series.

The cloud retrieval process starts with the determination of the cloud mask. Approaches based on statistical models and threshold based models have been proposed e.g., [4,5]. The operational cloud mask algorithms use threshold-based algorithms. The algorithms use a combination of spatial, temporal and spectral tests together with a set of thresholds to discern cloudy pixels from clear sky ones e.g., [6–13]. For example, the ABI Clear sky Mask (ACM) product uses a combination of the aforementioned tests with thresholds selected in such a way that no more than 2% of cloud over-detection (i.e., clear sky incorrectly classified as cloudy) is allowed [14].

In this study we quantify the performance of the GOES-R ACM product from the satellite located in the GOES East position, GOES-16, to detect clouds over the contiguous U.S. (CONUS). The main motivation is to assess the potential of this cloud mask product to initialize clouds in a NWP model designed for solar irradiance nowcasting called MAD-WRF. MAD-WRF combines the strengths of two models, MADCast [15] and WRF-Solar [16]. MADCast retrieves the cloud fraction from satellite infrared chanels and advects and diffuses this cloud fraction as a tracer using a modified version of the Weather Research and Forecasting model WRF, [17]. WRF-Solar is an augmented model specifically designed for solar energy applications and the main developments have focused on enhancing the aerosol-cloud-radiation interactions. The strength of MAD-WRF relies on the combination of the initialization process, wherein cloud retrievals can be assimilated, and the advection and diffusion of the initial hydrometeors as tracers to nudge the resolved hydrometeors at the beginning of the simulation, and, in this way, try to develop an appropriate environment to support the initial cloud field. Hence, an accurate cloud initialization is crucial for this particular application, and it is the motivation to assess the performance of the GOES-R ACM product.

The assessment is performed by comparing two years of the GOES-16 ACM retrievals interpolated to a 9 km grid covering the contiguous U.S. against the cloud mask retrievals from the Cloud-Aerosol Lidar and Infrared Pathfinder Satellite Observation CALIPSO, [18,19]. CALIPSO is equipped with an active sensor that to our knowledge provides the most accurate cloud detections covering CONUS during the multi-year period of analysis. The 9-km grid is well suited for nowcasting applications with MAD-WRF since it provides low latencies in production runs.

The multi-year assessment presented herein provides the most comprehensive evaluation of the ACM product over CONUS to date. The previous studies assessing the ACM performance that we are aware of have used also CALIPSO retrievals, but the studies have focused on a different region [14], or provided a basic evaluation of the product performance [5]. The algorithm theoretical basis [14] quantifies the performance of the ACM algorithm using retrievals from the Spinning Enhanced Visible and Infrared Imager (SEVIRI) on board of the Meteosat. Hence, the study does not include any retrieval over the CONUS. The assessment used eight weeks of data distributed over the four seasons and found a probability of detection, or accuracy (numer of correct retrievals divided by the number of retrievals), for all sky conditions (clear or cloudy) of 91.4%. The other study [5], focused on the development of an alternative retrieval algorithm based on statistical modeling, and only provides a basic evaluation of the ACM product as a baseline for comparison. Using about two months of data in each season, the study found an accuracy under all sky conditions of 85.7% with higher accuracy for cloud detection, 93.0%, than for clear sky detection, 67.7%. The study used the same grid under consideration herein, and thus the present work can be considered to a certain extent as an extension of the evaluation presented in [5]. The novel aspects of this study include a better pairing of GOES-16 and CALIPSO retrievals, and the use of a multi-year evaluation period. The multi-year period allows for a statistically robust characterization of the performance during daytime/nighttime using either the complete dataset or subsets for the four seasons for the first time. Additionally, using a multi-year period provides sufficient data to quantify the performance on spatial plots, and, to the best of our knowledge, this is the first time that the ACM performance have been characterized on two dimensional maps. Hence, we are able to identify the periods and regions wherein the ACM product shows its largest potential to initialize the clouds in the MAD-WRF model.

The manuscript is organized as follows. Section 2 describes the GOES-16 and CALIPSO retrievals as well as the spatio-temporal matching of both datasets. Section 3 presents the results, and the discussion is presented in Section 4.

## 2. Materials and Methods

### 2.1. Pairing GOES-16 and CALIPSO Retrievals

The GOES-R ACM product is described in [14] and it is herein briefly summarized. The product provides a binary clear-sky mask (clear or cloudy) every 10 min (full disk), at 2 km of horizontal resolution at the satellite subpoint. Data from from nine channels are used by the ACM algorithm. The algorithm is based on multiple spectral, spatial and temporal tests classified into four groups: infrared cloud detection tests, shortwave infrared cloud detection tests, solar reflectance cloud detection tests, and clear sky uniformity tests. The thresholds to discern clear from cloudy skies were selected using observations from the SEVIRI instrument on board the Meteosat collocated with CALIPSO retrievals. The assessment performed over the complete region covered by SEVIRI, during eight weeks distributed over the four seasons, shows a 91.4% of correct detections of the binary sky state (clear or cloudy) with 3.7% (4.9%) false cloud (clear) detection. The interested reader is referred to [14] for further details of the algorithm.

**Figure 1.**
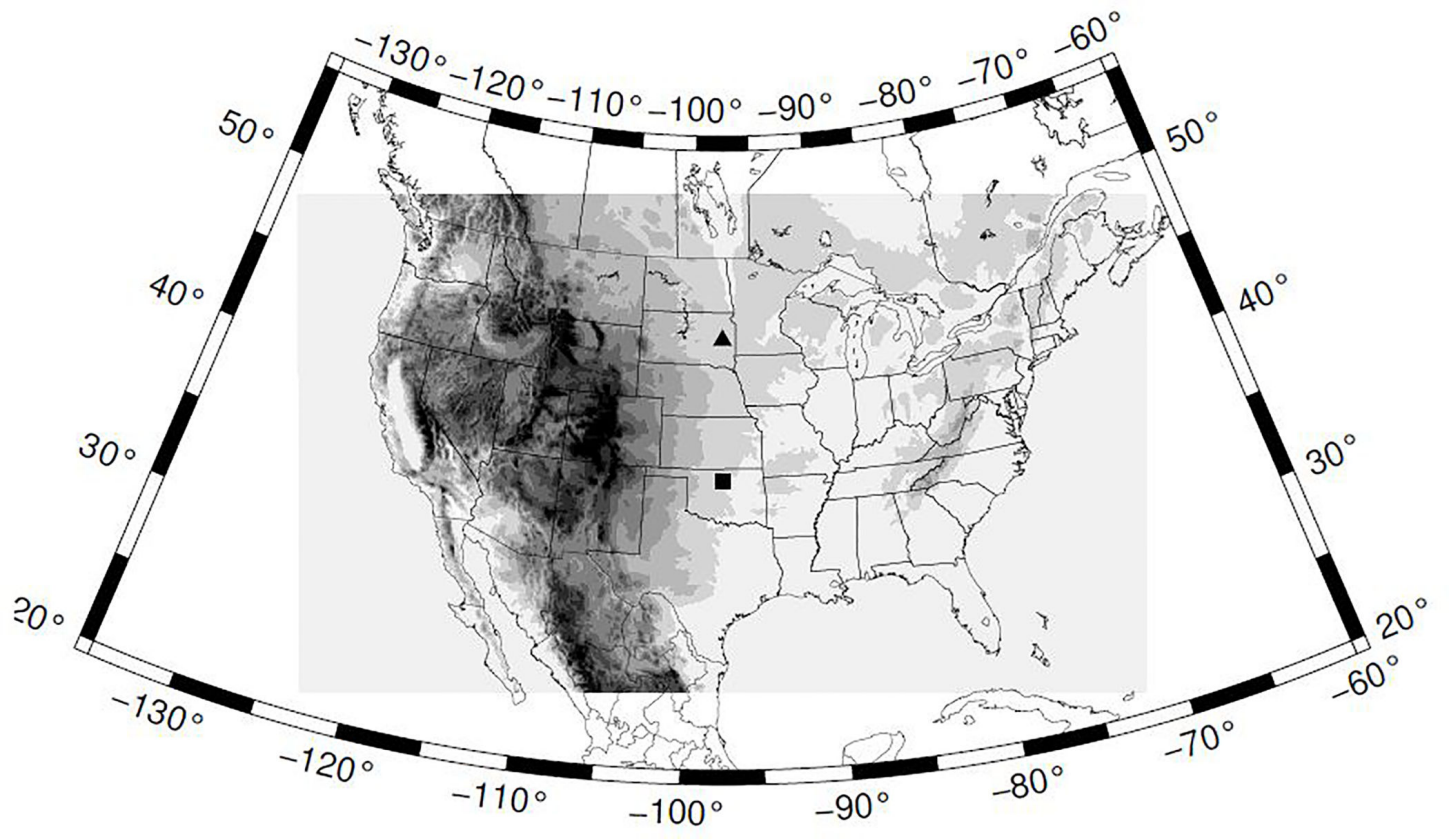
Region covered by the 9 km grid. A rectangle and a triangle mark grid rows with latitudinal changes in the ACM performance discussed in this work. Elevation is also shown (shaded).

The performance of the GOES-16 ACM product is quantified using CALIPSO cloud retrievals over the CONUS spanning the years of 2018 and 2019. The CALIPSO cloud retrieval starts by finding atmospheric features with the selective, iterated boundary locator (SIBYL) algorithm [19]. The features detected by the SIBYL algorithm correspond to regions of enhanced attenuated backscatter profiles. The algorithm also provides optical and physical properties of the features including the top and base heights. Based on these properties, each feature is classified by the scene classification algorithms [20]. One component of this algorithm is the cloud-aerosol discrimination (CAD) algorithm [21,22]. The CAD algorithm is a multi dimensional probability density function approach to discern between cloud and aerosol layers. The clouds are characterized by the CALIPSO cloud layer product at 1 km resolution (validation stage 1, version 3.40). A CALIPSO retrieval is considered cloudy when a cloud top height is reported and the CAD score is higher than 30. After identifying the binary clear-sky mask in a given CALIPSO pass over the CONUS, the horizontal cloud fraction over the 9 km target grid (Figure 1) is calculated by averaging the retrievals within each grid cell. A minimum of two CALIPSO retrievals within a grid cell is imposed to calculate the cloud fraction. The cloud fraction is subsequently converted into a binary cloud mask. If the cloud fraction is zero the grid cell is classified as clear whereas if the cloud fraction is larger than zero it is classified as cloudy. No error is considered in the cloud mask retrieval. This is motivated by the quality of the CALIPSO retrievals, and by the averaging process to transform the binary cloud mask into the horizontal cloud mask that is expected to minimize the impact of CALIPSO miss retrievals. Next, we associate a time stamp to the cloud mask grid. The CALIPSO passes over the CONUS are fast, typically around 10 minutes, and thus the grid is assigned the midtime between the time the satellite entered the CONUS and the time the satellite left the CONUS.

Each cloud fraction field from the CALIPSO passes over the CONUS is associated with an ACM retrieval if possible. For this purpose, we first assign to each full disk ACM retrieval the approximated time of the data retrieved over the CONUS. Considering that ABI starts the scan from the top of the disk, and is at the equator at the mid time between the starting and ending time of the scan, the time associated with the data over CONUS is estimated as the starting time plus one eighth of the time it takes to complete a scan. If an ACM scan is within 600 s of the CALIPSO pass over CONUS we consider this a match and the ACM product is used to calculate the horizontal cloud fraction in the 9 km grid. Similarly for CALIPSO, this is accomplished by averaging the binary cloud mask within each grid cell. Finally, the binary cloud fraction is calculated assigning clear sky if the cloud fraction is zero and cloudy otherwise.

**Figure 2.**
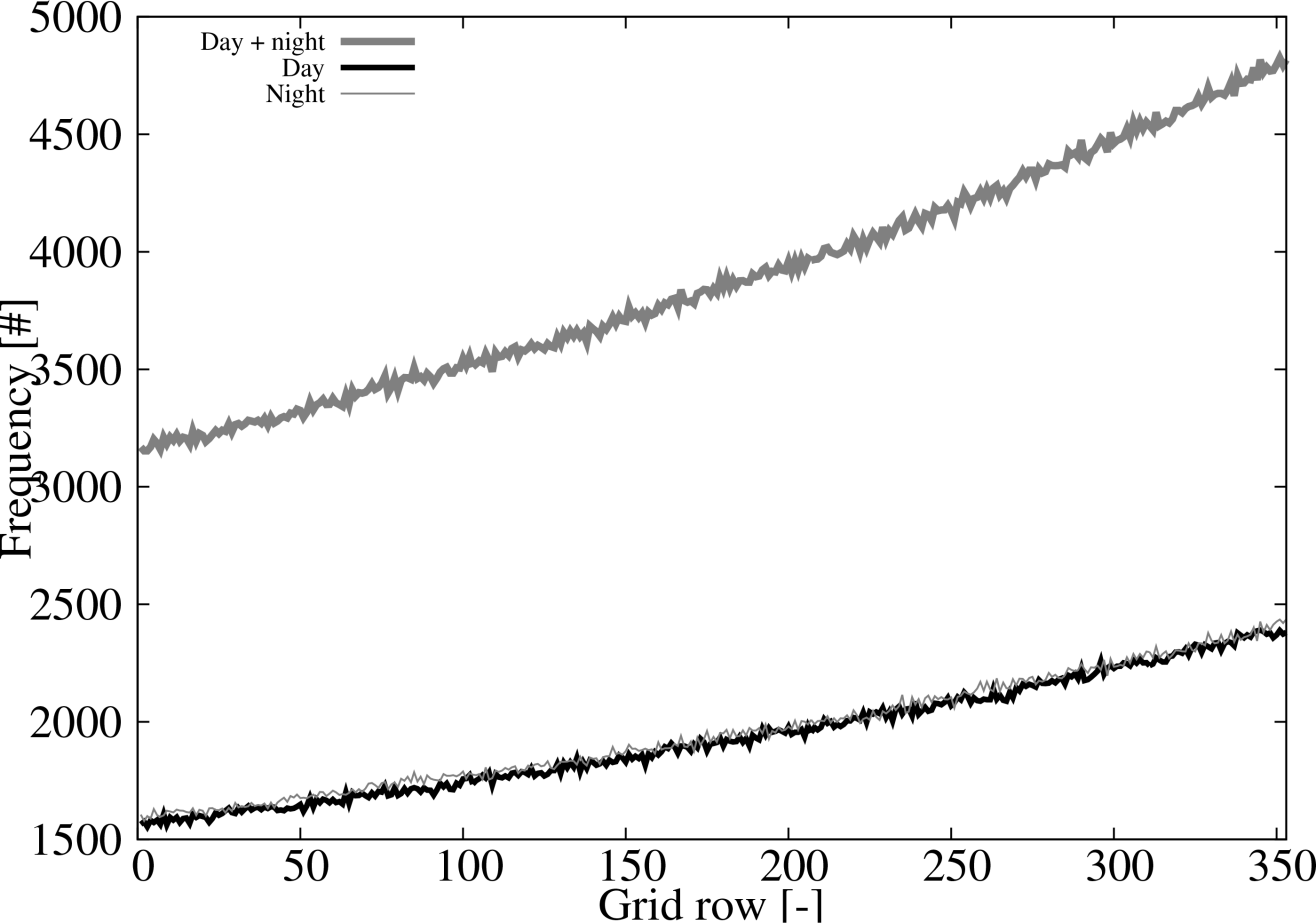
Number of CALIPSO and ACM matching pairs as a function of the grid row for: day and night pairs (thick gray ine), daytime pairs (black line), and nighttime pairs (thin gray line).

The set of pairs of binary cloud masks (1371012 grid points) are used to evaluate the ACM performance. The number of pairs as function of the grid row is shown in Figure 2. The number of pairs increases as the row number increases because CALIPSO is a circumpolar satellite. There are over 3000 pairs in the first grid row and almost 5000 pairs in the last one (thick gray line). The distribution of pairs is very close for day (black line) and night (thin gray line) which is a desirable characteristic for the evaluation.

### 2.2. Evaluation

The primary emphasis on the evaluation is herein placed on the probability of detection or accuracy. This is defined as the number of correct retrievals divided by the total number of retrievals. Three accuracies are analyzed: (1) the accuracy of the cloud mask for all sky conditions; (2) the accuracy of the cloud detection; and (3) the accuracy of the clear sky detection. These set of accuracies are calculated with the whole set of matched pairs and with subsamples to characterize the performance of the ACM product as a function of the season, day/night, and latitude.

To gain further physical insight of the ACM performance, the evaluation also examines the performance in terms of the cloud top height. With this aim we use both CALIPSO cloud top height retrievals and the cloud top height retrievals from the ABI Cloud Height Algorithm ACHA, [23]. The cloud top height from CALIPSO is used to inspect the height of the cloud misses. For these purposes, the cloud top height retrievals are averaged to the 9 km grid in an analogous way to the cloud fraction. On the other hand, the ACHA product is used to inspect the cloud top height of the clear sky misses. This product is not available for large satellite zenith angles which causes a lack of retrievals in the northwest portion of our grid. Furthermore, although the ACHA product is provided every full disk scan, the cloud top height is provided over a grid with a 10 km grid spacing at the satellite subpoint. Hence, in order to have retrievals over our target grid, we assign the nearest grid point from a given ACHA retrieval to the 9 km grid cells. This will produce less accurate cloud top height retrievals than the cloud mask but the data are still useful to investigate the cloud height of the clear sky misses.

## 3. Results

**Figure 3.**
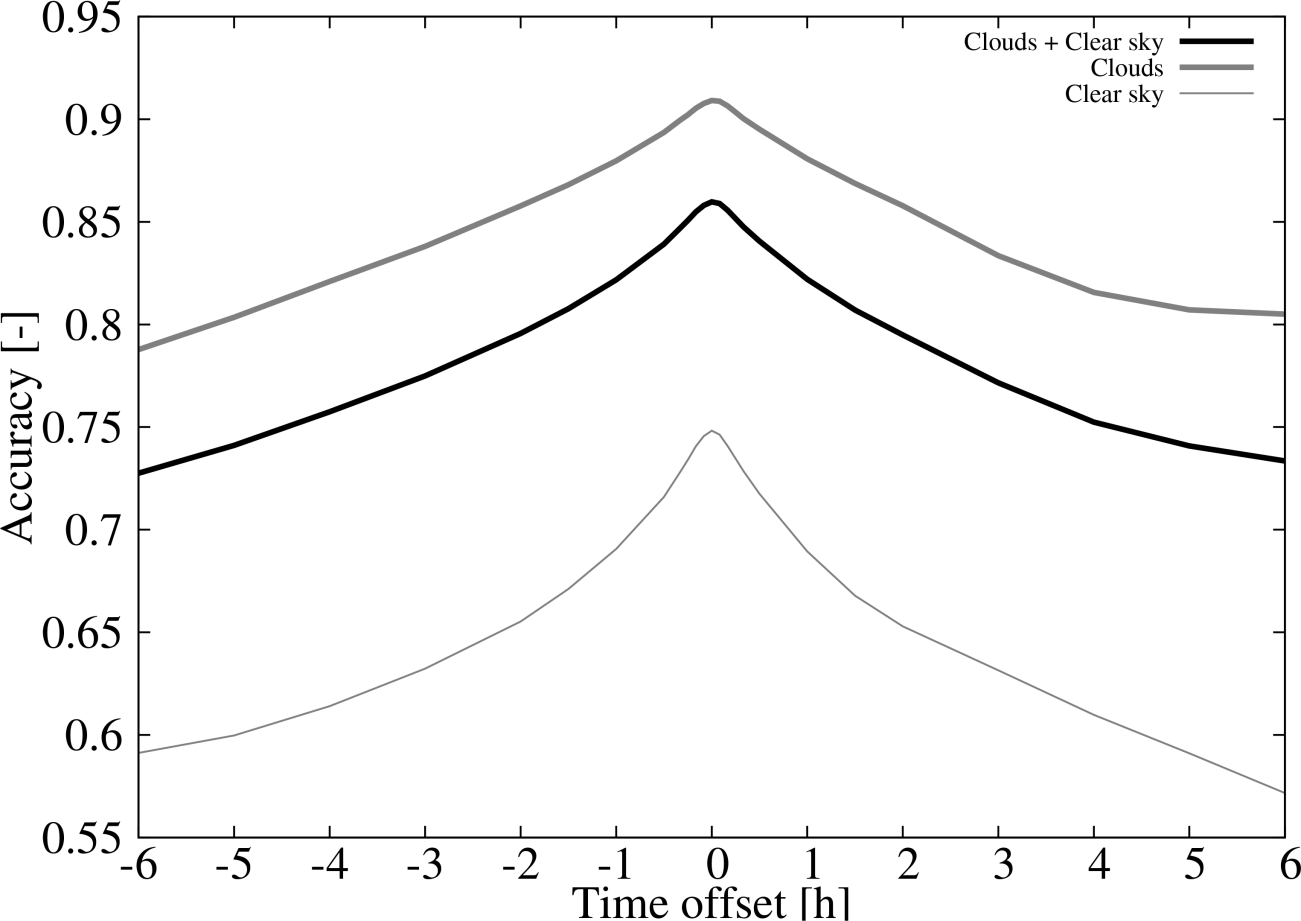
Accuracy of the (1) clouds and clear sky detection (black line), (2) cloud detection (gray thick line), and (3) clear sky detection (gray thin line) as a function of the time offset.

The accuracy of the ACM product for all sky conditions calculated with the two years of retrievals during 2018 and 2019 is 86.0%. However, the accuracy for the cloud detection is higher, 90.9%, than the clear sky detection, 74.8%. To assess the impact of the temporal matching of CALIPSO and ACM retrievals, the set of accuracies as a function of a time offset are shown in Figure 3. As expected, the curves show the maximum when no offset is introduced. When the temporal offset is introduced, the accuracy decays at a faster pace for the clear sky detection than for the cloud detection. For instance, for a 1 h time offset the accuracy of the cloud detection drops to 88.0% (2.3% reduction) whereas for the clear sky drops to 69.0% (7.7% reduction). This is a consequence of having more clouds (950350, 69.3%) than clear sky (420662, 30.7%) retrievals and probably an over-detection of clouds by the ACM product.

**Figure 4.**
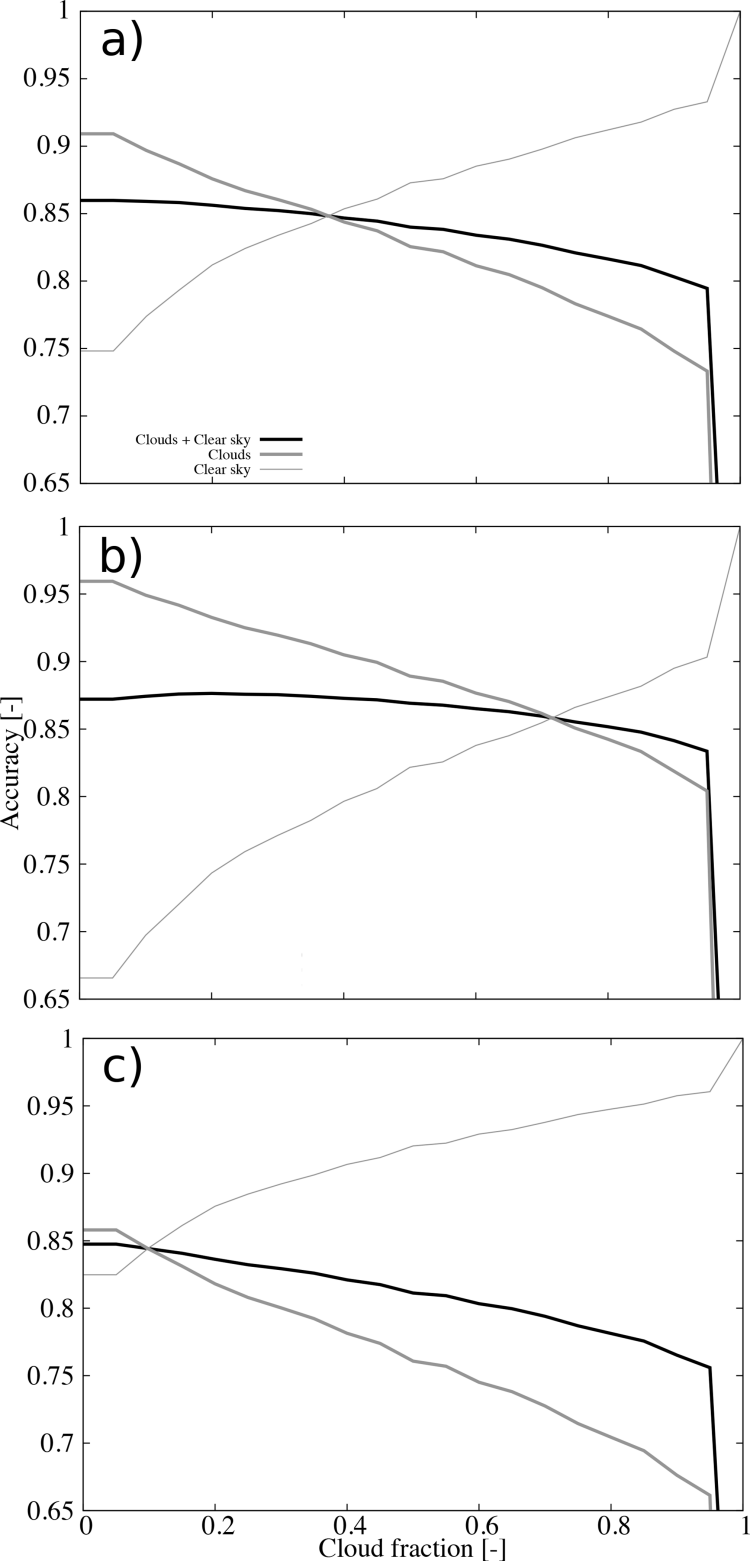
Accuracy as a function of the cloud fraction threshold for (**a**) all retrievals, (**b**) daytime retrievals, and (**c**) nighttime retrievals. The accuracy of the clouds and clear sky detection, cloud detection, and clear sky detection are shown (see legend).

The amount of clouds in our grid is modulated by the cloud fraction threshold used to create the binary cloud mask from the cloud fraction calculated with the ACM product. This threshold has been set to zero, and there is not a solid theoretical basis to change it, but analyzing sensitivities to this threshold will help us understand the performance of the ACM product. With this aim, the accuracy as a function of the cloud fraction threshold is shown in Figure 4a. As expected, the accuracy of the cloud detection decreases as the cloud fraction threshold increases whereas the opposite is true for the clear sky detection. The accuracy for all sky conditions shows the maximum for a zero cloud fraction threshold which is also the expected behavior (and in agreement with [5]). However, the behavior of the accuracy metrics differs for daytime and nighttime (Figure 4b,c). During daytime (Figure 4b), the discrepancies already noticed in the cloud and clear sky detections are exacerbated. For a zero cloud fraction threshold, the accuracy for cloud detection is 95.9% whereas clear sky detection is 66.6%. Interestingly, the performance of all sky conditions does not show the maximum for a zero cloud threshold (87.2%) but at 0.20 (87.6%), but the curve is rather flat. The high (low) accuracy for cloud (clear sky) conditions suggests a cloud over-detection during daytime. On the contrary, nighttime shows similar accuracy for cloud and clear sky detections (85.8% and 82.5%, respectively) for a zero cloud fraction threshold (Figure 4c). In addition, the maximum of the accuracy for all sky conditions is at a zero cloud fraction (84.7%).

**Figure 5.**
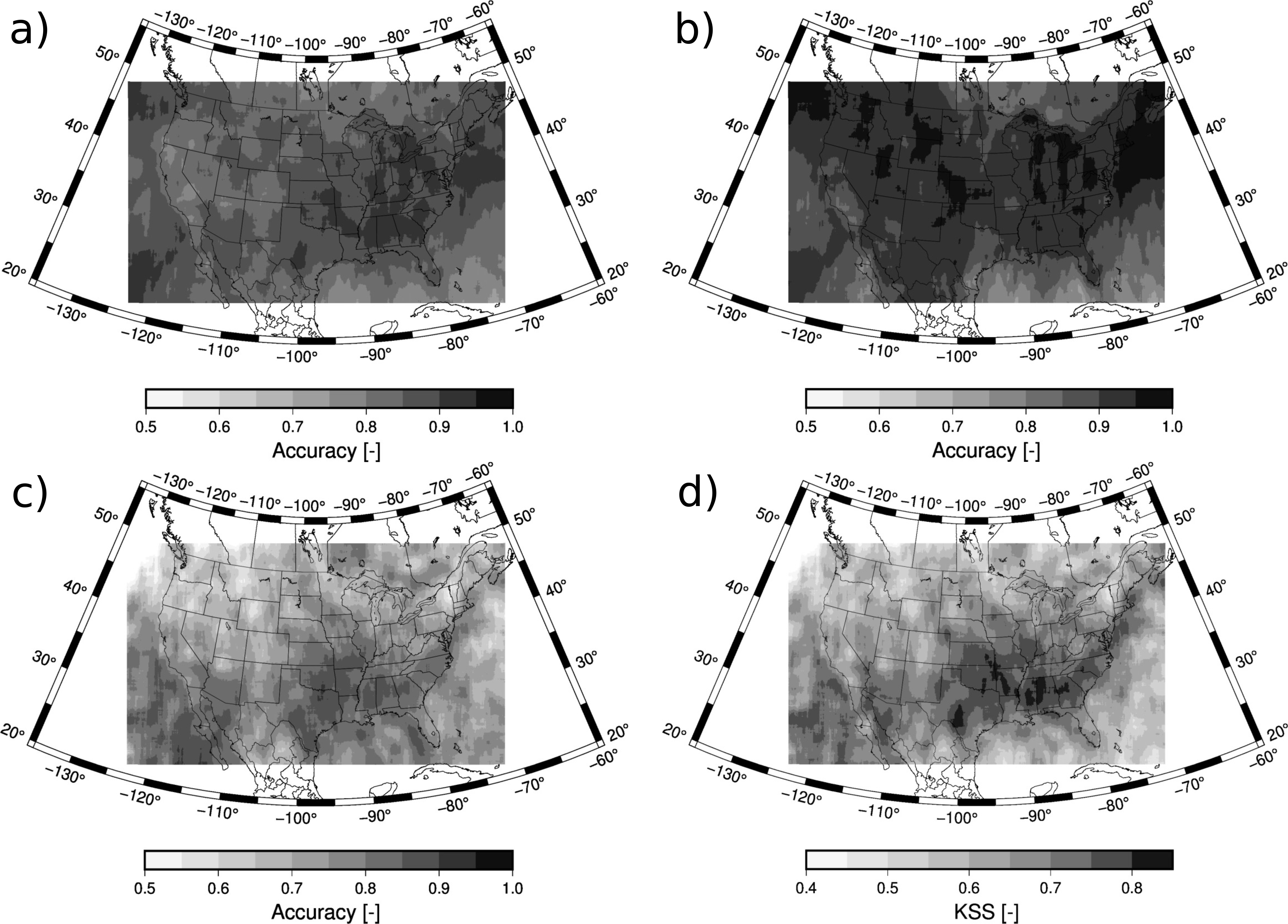
Accuracy for (**a**) all sky retrievals, (**b**) cloud detection, and (**c**) clear sky detection. The KSS is also shown (**d**). The values are calculated using data within 15 by 15 grid points.

Figure 5 shows the spatial distribution of the accuracy. Results for each grid cell are calculated with the matched pairs of ACM and CALIPSO cloud mask over a region of 15 by 15 grid points. This smoothing is necessary since, despite using two years of data, the spatial density is not sufficient to display results at 9 km of grid spacing. The accuracy for all-sky conditions is higher for the eastern part of the CONUS wherein the accuracy is larger than 90% (Figure 5a). The western portion of the CONUS shows lower values but never smaller than 70%. As already pointed out, the accuracy is larger for the cloud detection (Figure 5b) than for the clear sky detection (Figure 5c). More specifically, the accuracy is larger than 90% almost everywhere in the CONUS, being larger than 95% in certain regions. On the other hand, the clear sky accuracy shows the largest values in the south of the CONUS, larger than 85%, but some regions in the north show accuracies close to 50%. Low accuracy values over the Rocky Mountains can also be appreciated. In spite of this different behavior between clear skies and cloudy skies, the higher frequency of the later leads to an all-sky accuracy more influenced by the results of the cloud detection. A skill score that takes into account the frequency of both populations is the Kupier’s skill score (KSS). KSS is defined as the difference between the hit rate and the false alarm rate and it ranges from −1 to 1 with 0 indicting no skill. The distribution of the KSS is shown in Figure 5. Over the CONUS, the pattern is more similar to the clear sky accuracy (Figure 5c) since the accuracy of the cloud detection is very high (Figure 5b). The KSS shows values larger than 0.8 in the south and lower values in the north that can reach down to 0.4.

**Figure 6.**
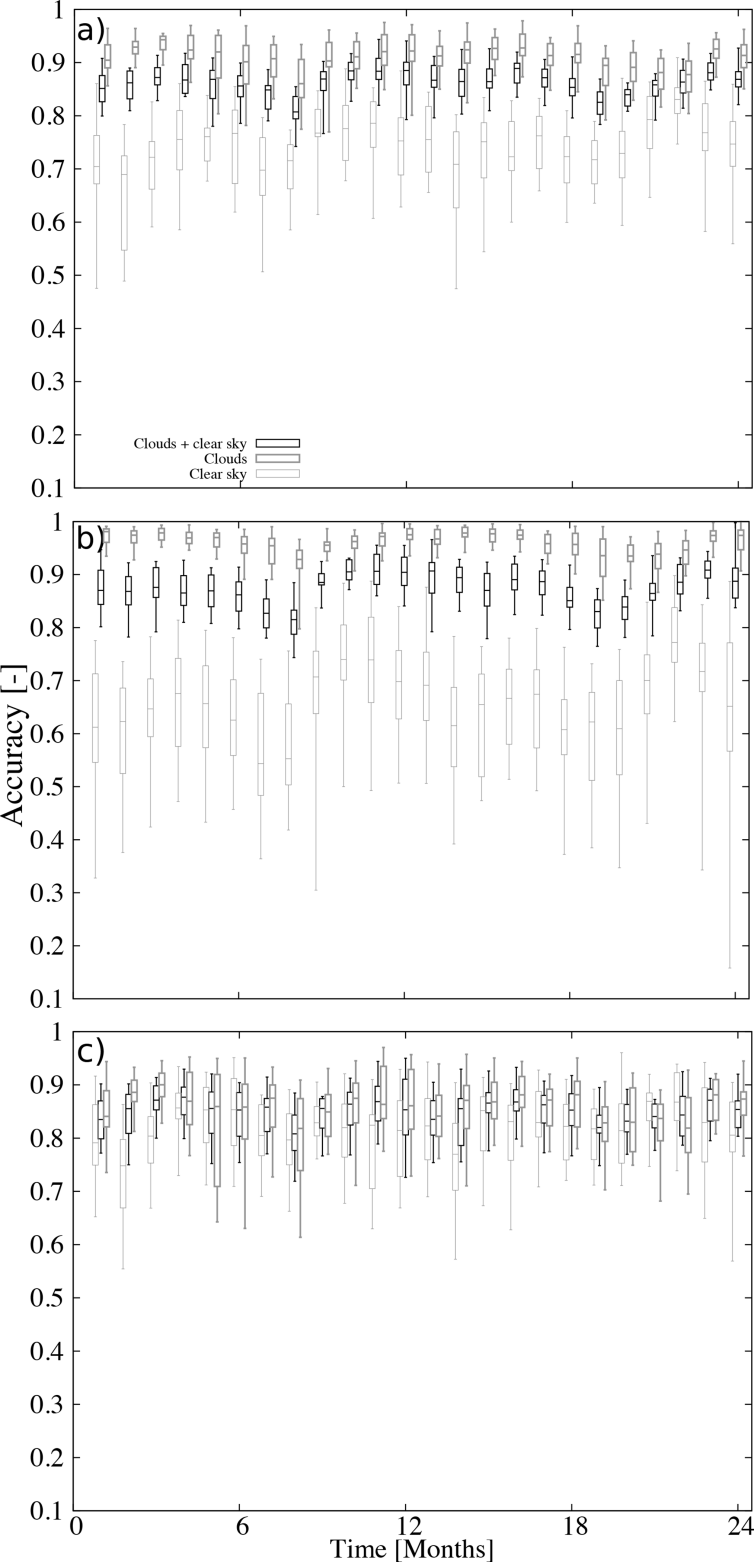
Box and whiskers plots of the daily accuracy on a motnthly basis for (**a**) all retrievals, (**b**) daytime retrievals, and (**c**) nighttime retrievals. The middle bar indicates the median, the lower (upper) bases of the boxes are the 25 (75) percentiles, and the lower (upper) whiskers indicate the 5 (95) percentiles. The accuracy for all-sky conditions, cloud detection, and clear sky detection are shown (see legend).

The evolution of the accuracy over the two year period is shown in Figure 6 that shows the box and whiskers plots of the daily accuracy on a monthly basis. The median of the accuracy (middle bar) for all sky conditions has small variability with a tendency to show the lowest values during the summer (Figure 6a). This is also evidenced during daytime (Figure 6b) and it is less clear during nighttime (Figure 6c). The largest variability is for the clear sky detection during daytime (Figure 6b) that, in addition to the minimum during the summer, shows a maximum during the fall. The range of variability is about 20%. On the contrary, the median of the accuracy of nighttime retrievals shows low variability over the year (Figure 6c). The monthly distributions during daytime (Figure 6b) are wider for clear sky detections than for clouds or all sky conditions. However, this behavior is not recognized during nighttime (Figure 6c) wherein the inter-quantile distance (percentile 75 minus percentile 25) is similar for both clouds and clear sky detections.

**Figure 7.**
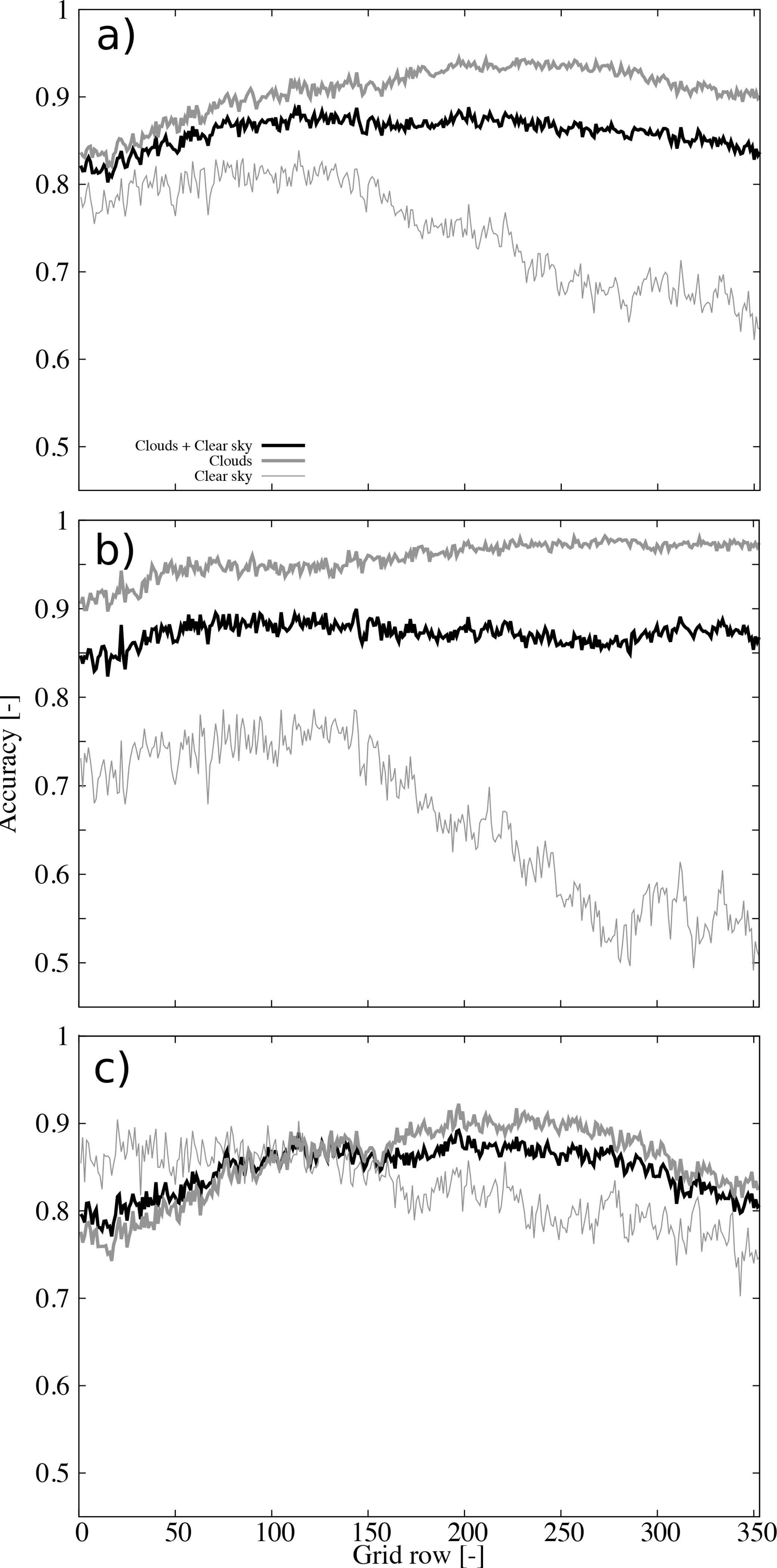
Accuracy as a function of the grid row for (**a**) all retrievals, (**b**) daytime retrievals, and (**c**) nighttime retrievals. The accuracy for all-sky conditions, cloud detection, and clear sky detection are shown (see legend).

Further insights on the performance become evident analyzing the accuracy as a function of the grid row (Figure 7). The accuracy under all sky conditions is higher than clear skies for all rows, or latitudes, under consideration (Figure 7a). For clear sky retrievals there is a clear degradation of the accuracy for the rows higher than about 150 (square in Figure 1, roughly parallel 36). These findings were already recognized in the spatial patterns of the accuracy (Figure 5). The clear sky degradation as a function of latitude is a consequence of the daytime retrievals (Figure 7b) since the accuracy of the nighttime retrievals shows smaller variations with latitude (Figure 7c). The accuracy of daytime retrievals beyond grid row 250 (triangle in Figure 1, roughly parallel 44) is very close to 0.5 which suggests small skill of the cloud mask in this subsample.

**Figure 8.**
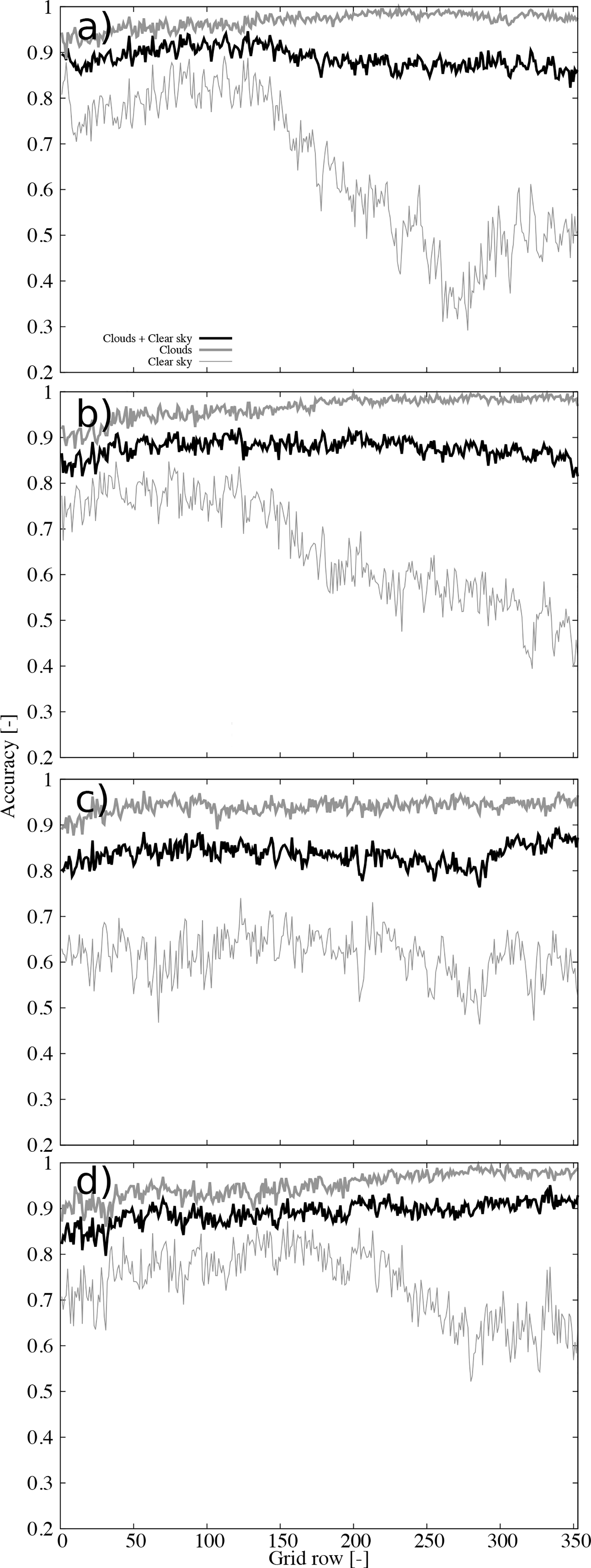
Accuracy as a function of the grid row for daytime during (**a**) Winter, (**b**) Spring, (**c**) Summer, and (**d**) Autumn. The accuracy for all sky conditions, cloudy, and clear sky are shown (see legend).

**Figure 9.**
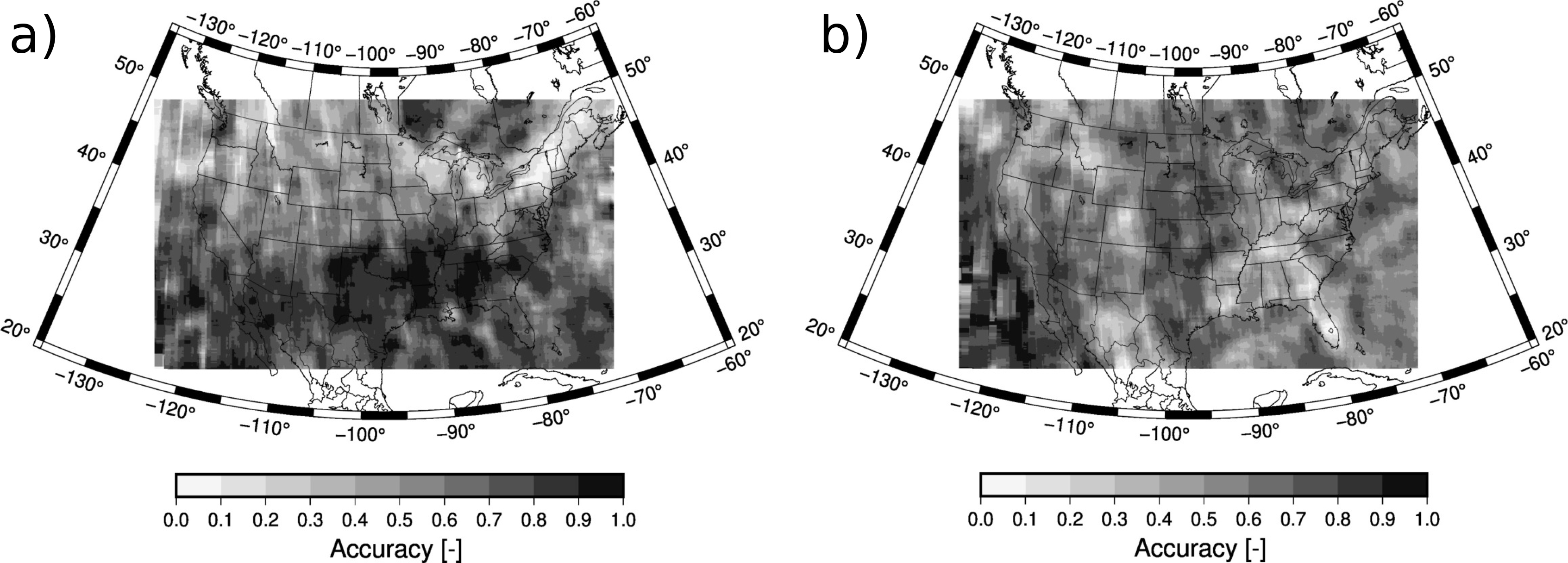
Accuracy for the daytime clear sky retrievals during (**a**) Winter and (**b**) Summer. The values are calculated using data within 15 by 15 grid points.

To further examine the origin of these uncertainties, Figure 8 shows the accuracy during daytime for the four seasons. Clearly, the erroneous retrievals dominating the previous behavior occur during winter (Figure 8a). The spatial distribution (Figure 9a) indicates this is a general behavior over the CONUS, with larger latitudinal contrast in the east. The summer season (Figure 8c) shows little dependence on the latitude, although the accuracy is somewhat low (around 0.6). The spatial distribution reveals large spatial variability during this season (Figure 9b). The Spring and Autumn (Figure 8b,d) show intermediate behavior between the winter and summer seasons.

**Figure 10.**
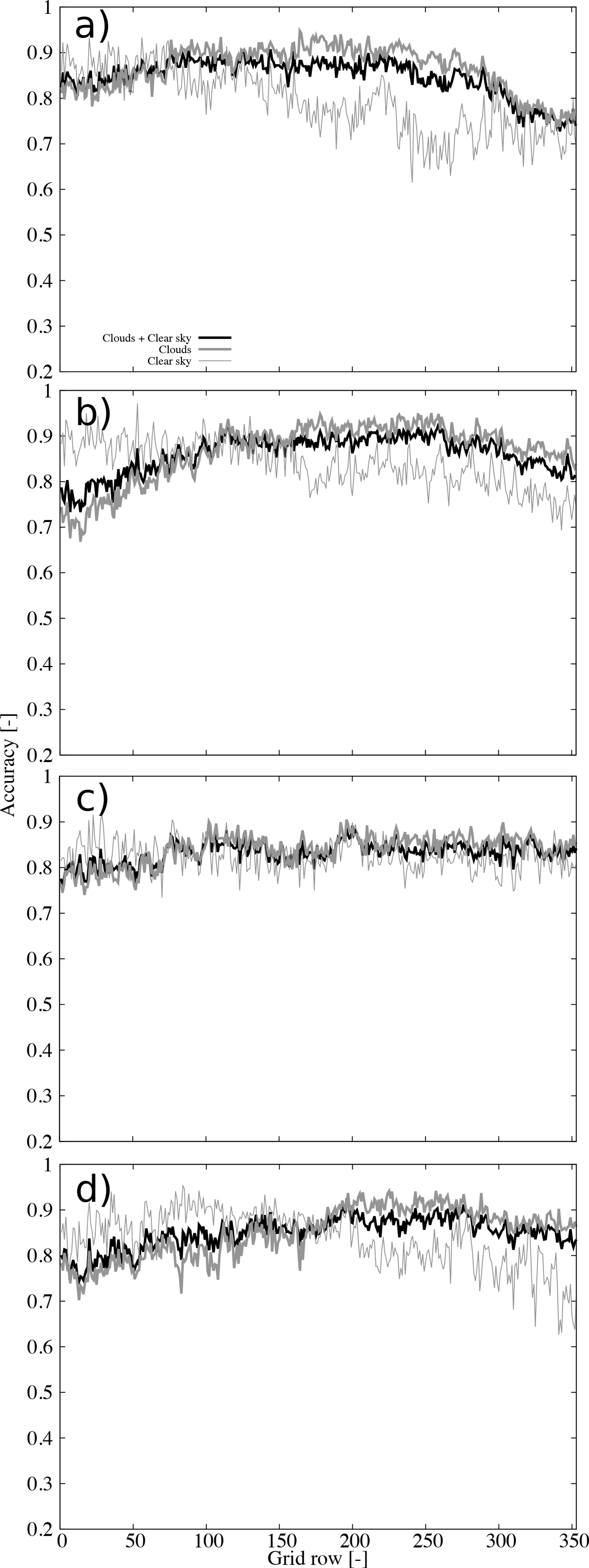
Accuracy as a function of the grid row for nighttime during (**a**) Winter, (**b**) Spring, (**c**) Summer, and (**d**) Autumn. The accuracy for all sky conditions, cloudy, and clear sky are shown (see legend).

Nighttime conditions show small variability as a function of the latitude during the four seasons (Figure 10), with remarkable similar behavior (accuracy generally higher than 0.8) of the cloud and clear sky retrievals during summer (Figure 10c).

**Figure 11.**
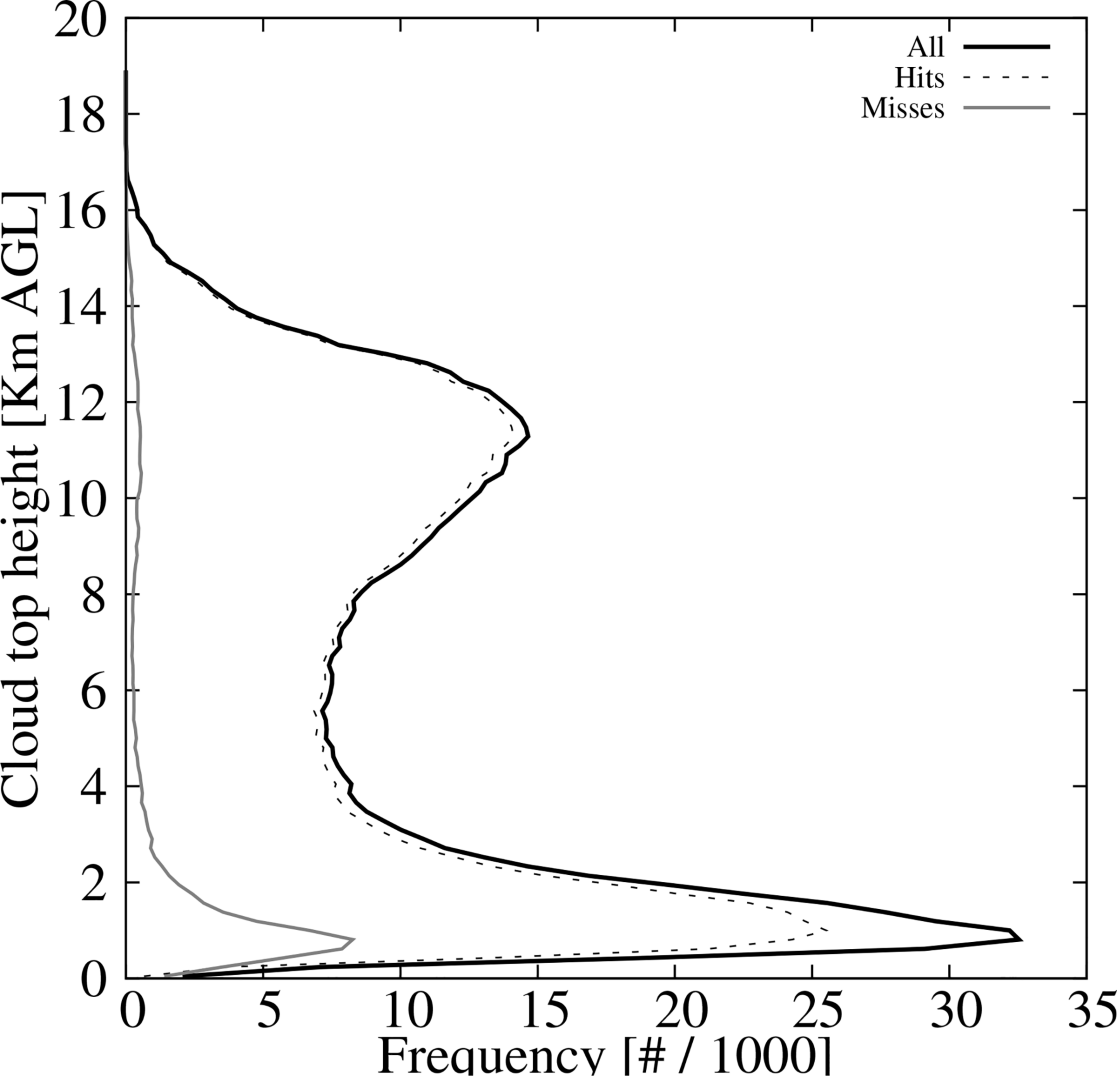
CALIPSO cloud top height histogram for all retrievals, (black line), cloud hits (black dashed line), and cloud misses (gray line).

To better understand the uncertainties of the ACM product, the cloud top height histogram from the CALIPSO retrievals is shown in Figure 11. The distribution has two maxima (black solid line) one around 1 km above ground level (AGL), boundary layer clouds, and the other at around 12 km AGL, clouds at the top of the troposphere. Figure 11 also shows the histogram of the cloud hits/misses which clearly shows a missdetection of boundary layer clouds. This pattern of underestimating low clouds is found during both day and night retrievals as well as in the four seasons (not shown).

In order to understand the origin of the missdetections under clear sky conditions we use the ACHA product. First, we inspect the accuracy of the binary cloud mask calculated with the ACHA product. The all sky accuracy is 82.9 %. The accuracy of the cloud retrievals is 91.2% whereas the clear sky detection is 64.8%. These values are similar to the ones obtained with the ACM product but there is a decay in the clear sky detection (13.4%). This is partially related to the different spatial resolution of the ACHA product (10 km) with respect to the ACM product (2 km), with probably a small modulation due to the reduced spatial coverage of the ACHA product.

**Figure 12.**
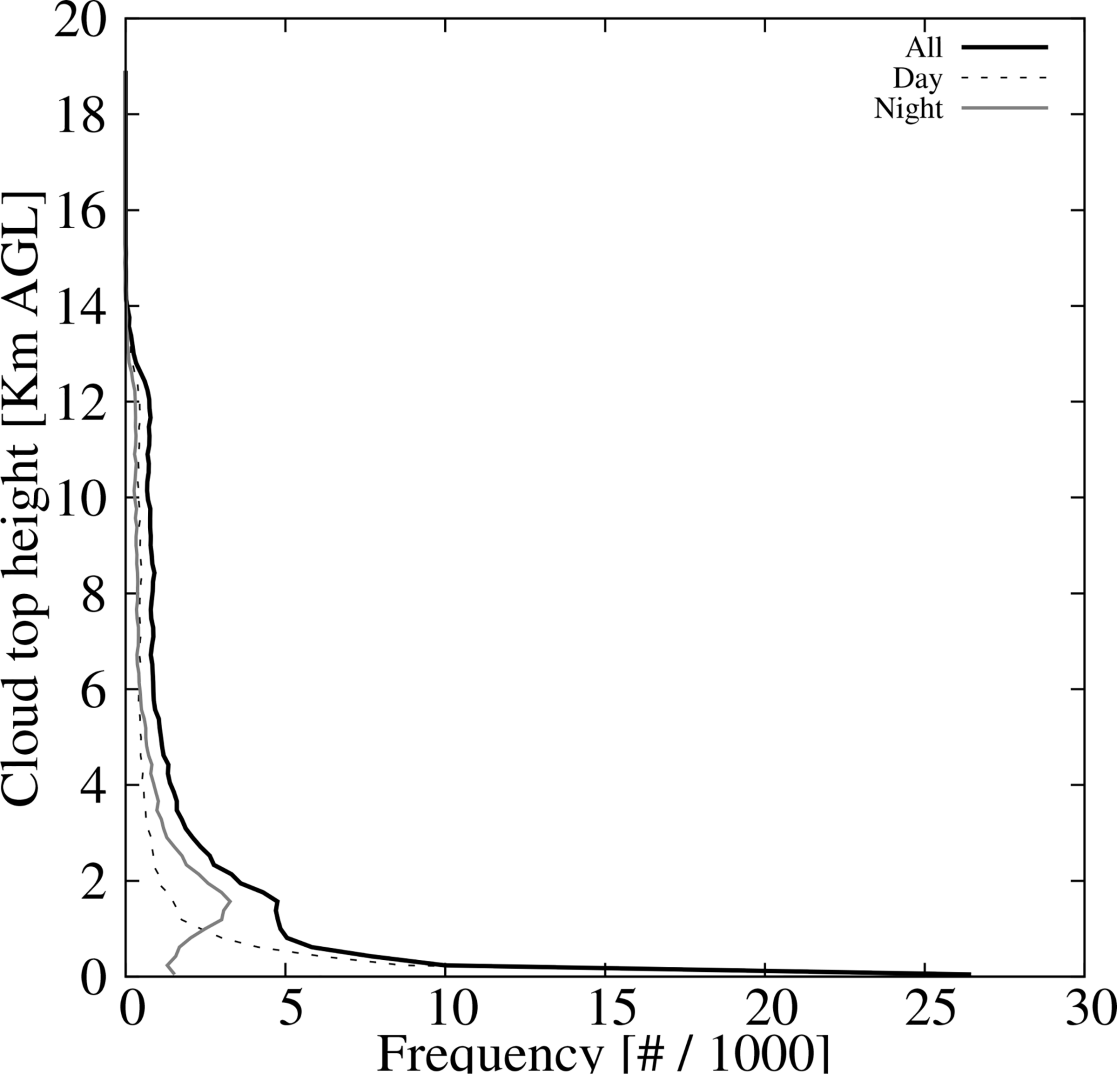
Histogram of the cloud top height for the over-detected clear skies from the ACHA product for both day and night retrievals (black solid line), daytime retrievals (black dashed line), and nighttime retrievals (gray solid line).

The histograms of the cloud top height retrievals from the ACHA product for those cases missclassified as clear sky are shown in Figure 12. The distribution shows the maximum near the surface and a secondary one at about 2 km AGL (black line). These peaks are mainly associated with daytime retrievals (dashed black line) and nighttime retrievals (gray line). The daytime maxima near the surface is present during the four seasons whereas the nighttime maxima mostly occur during the summer (not shown). As with the cloud missdetections, the main discrepancies are associated with low level clouds.

## 4. Discussion

The performance of the ACM product over CONUS has been examined. The evaluation has been performed comparing the binary cloud mask on a 9 km grid to CALIPSO retrievals on the same grid. Two years of retrievals have been used to provide a statistically robust characterization of the product. The probability of detection or accuracy under all sky conditions is 86.0% being the accuracy of cloud detection higher, 90.9 %, than the clear sky detection, 74.8%. The all sky detection and cloud detection are in agreement with [5] that found an accuracy of 85.7% and 93.0%, respectively. The clear sky detection is larger than the 67.7% reported in [5]. The values found in this evaluation should be more accurate considering the much longer evaluation period, and the better temporal match of observations ([5] used the completion time of the retrievals for ACM and the nearest hour for the CALIPSO retrievals, allowing for 800 s differences between both time stamps). The lower performance of clear sky retrievals is a result of missdetections during daytime. This is especially clear for summer, and for regions to the north of parallel 36 during winter. The nighttime retrievals show similar performance of the cloud and clear sky retrievals.

The majority of the missdetections are associated with low clouds. The undetected clouds generally have the cloud top height within the first 2 km AGL, with the maximum around 1 km AGL. This is in agreement with [14] findings over the region covered by the Meteosat. The cloud over-detection is also associated with low level clouds. In this case, there is a different behavior during daytime and nighttime. During daytime, the cloud top height distribution of the clouds overdetected shows a maximum near the ground and a rapid decrease with height. There are fewer overdetections for cloud top heights higher than 2 km. During nighttime, the maximum of the cloud top height distribution is around 2 km.

The main motivation for this assessment was to evaluate the potential of the ACM product for initializing the clouds in the MAD-WRF NWP model. MAD-WRF is a solar irradiance nowcasting system and thus it is important to have an accurate cloud initialization, especially during daytime. However, the ACM product shows superior performance during nighttime than during daytime. The performance of daytime retrievals to the north of parallel 36 is probably not adequate for this particular application. However, regions to the south of parallel 36 show acceptable performance during both daytime and nighttime. It is over these regions wherein the ACM product should show its largest potential to enhance the cloud initialization in the MAD-WRF model.

**Figure 13.**
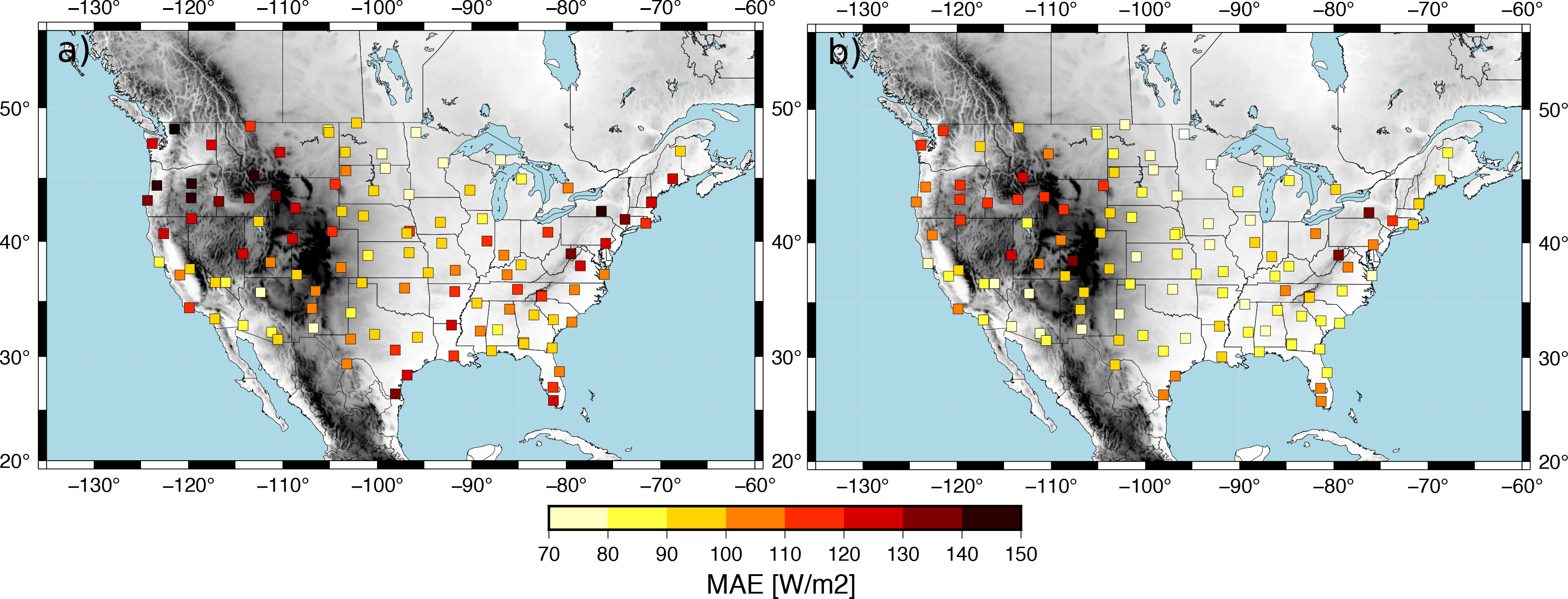
MAE of the GHI at the USCRN sites calculated using hourly analyses spanning the month of April 2018 for: (**a**) WRF-Solar and (**b**) MAD-WRF.

To assess these conclusions, Figure 13 shows the MAE calculated with global horizontal irradiance (GHI) hourly analyses performed for the month of April 2018 with both WRF-Solar and MAD-WRF. The GHI analysis was created using the 1 h forecasts from the High Resolution Rapid Refresh HRRR, [24] model run operationally by the National Center for Environmental Prediction (NCEP). The 1 h forecasts is available about 20 min before its valid time which allows one to use it in nowcasting applications. Comparison is performed against the observations from the United States Climate Reference Network USCRN, [25]. The WRF-Solar model shows MAE values larger than 100 W m^−2^ at most of the sites with values larger than 150 W m^−2^ at certain locations (Figure 13a). These errors are reduced with the MAD-WRF analysis that imposes the GOES-16 cloud mask (Figure 13b). The MAD-WRF analysis shows MAE values lower than 100 W m^−2^ at the majority of the sites to the south of parallel 36. To the north of this parallel, the errors are higher for the western part of the CONUS, wherein topography is more complex, and in the eastern part. The errors are lower in the center of CONUS. Although we are using only one month of data, the spatial distribution of the MAE is in good agreement with the KSS score of the ACM product (Figure 5d). This further stresses the importance of having an accuracte retrieval to initialize the cloud field. Potential improvements in the performance of the GOES-16 ACM product should translate into a more accurate cloud initialization and thus an improved performance of the MAD-WRF model nowcasts.

## Supplementary Material

data_fig13

## Abbreviations

ABI: Advanced Baseline Imager
ACHA: ABI Cloud Height Algorithm
ACM: ABI Clear sky Mask
AGL: above ground level
CAD: Cloud-aerosol discrimination
CALIPSO: Cloud-Aerosol Lidar and Infrared Pathfinder Satellite Observation
CONUS: Contiguous U.S.
GHI: Global horizontal irradiance
GOES: Geostationary Operational Environmental Satellites
HRRR: High Resolution Rapid Refresh
KSS: Kupier’s skill score
MADCast: Multi-sensor Advection Diffusion nowCast
MODIS: Moderate Resolution Imaging Spectroradiometer
NWP: numerical weather prediction
NCEP: National Center for Environmental Prediction
VIIRS: Visible Infrared Imaging Radiometer Suite
SEVIRI: Spinning Enhanced Visible and Infrared Imager
SIBYL: Selective Iterated Boundary Locator
WRF: Weather Research and Forecasting
